# Supplementary material for: MoVE identifies metabolic valves to switch between phenotypic states
Source: Nat Commun. 2018 Dec 14;9:5332. doi: 10.1038/s41467-018-07719-4 (PMC6294006; doi:10.1038/s41467-018-07719-4)
Supplement: Supplementary file 1 — Supplementary Information [file 41467_2018_7719_MOESM1_ESM.pdf]

Supplementary Information

MoVE identifies metabolic valves to switch between phenotypic states
Venayak et al.

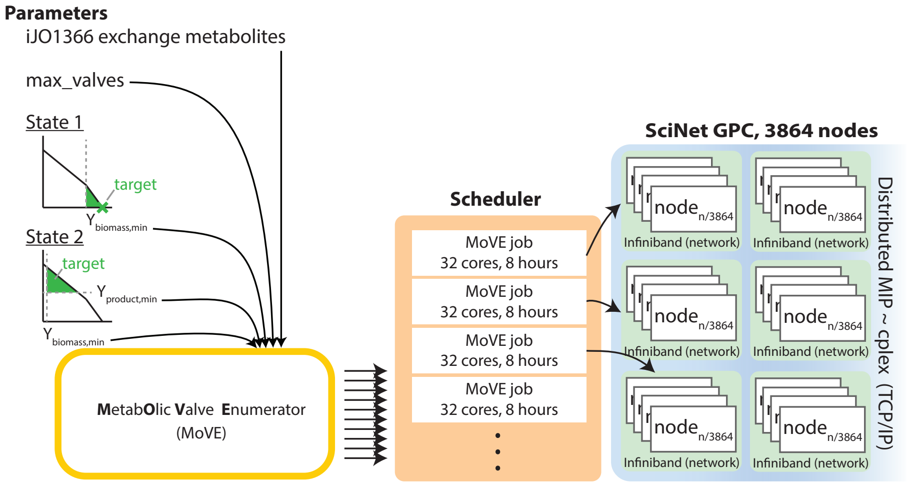

Supplementary Figure 1: Overview of distributed mixed-integer linear programming solution. Jobs are created for each metabolite and given simulation parameters, and solved on a general purpose cluster. Each job is solved for a total of 128 $core \cdot hours$ or 256 $thread \cdot hours$

.

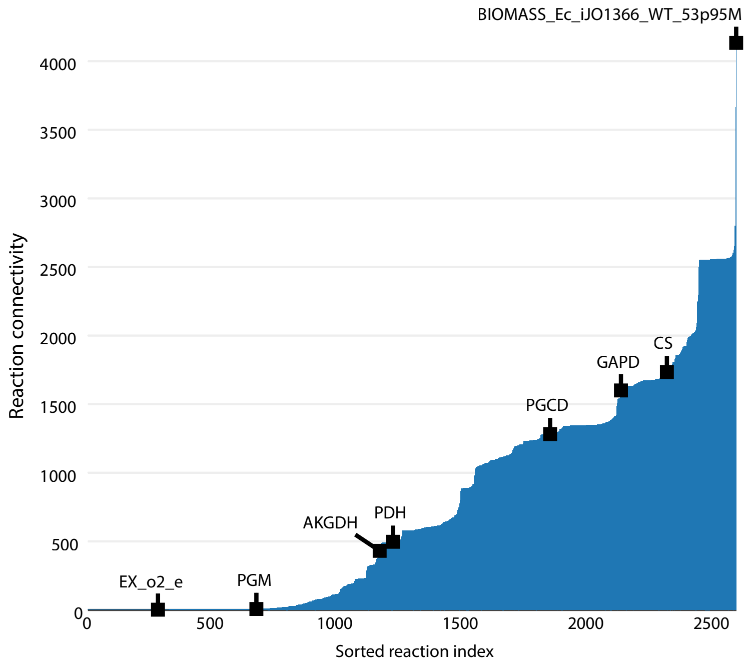

Supplementary Figure 2: Distribution of reaction connectivities. The connectivity of a given reaction is defined as the total number of reactions connected to all metabolites in that reaction. The reactions are sorted in ascending order.

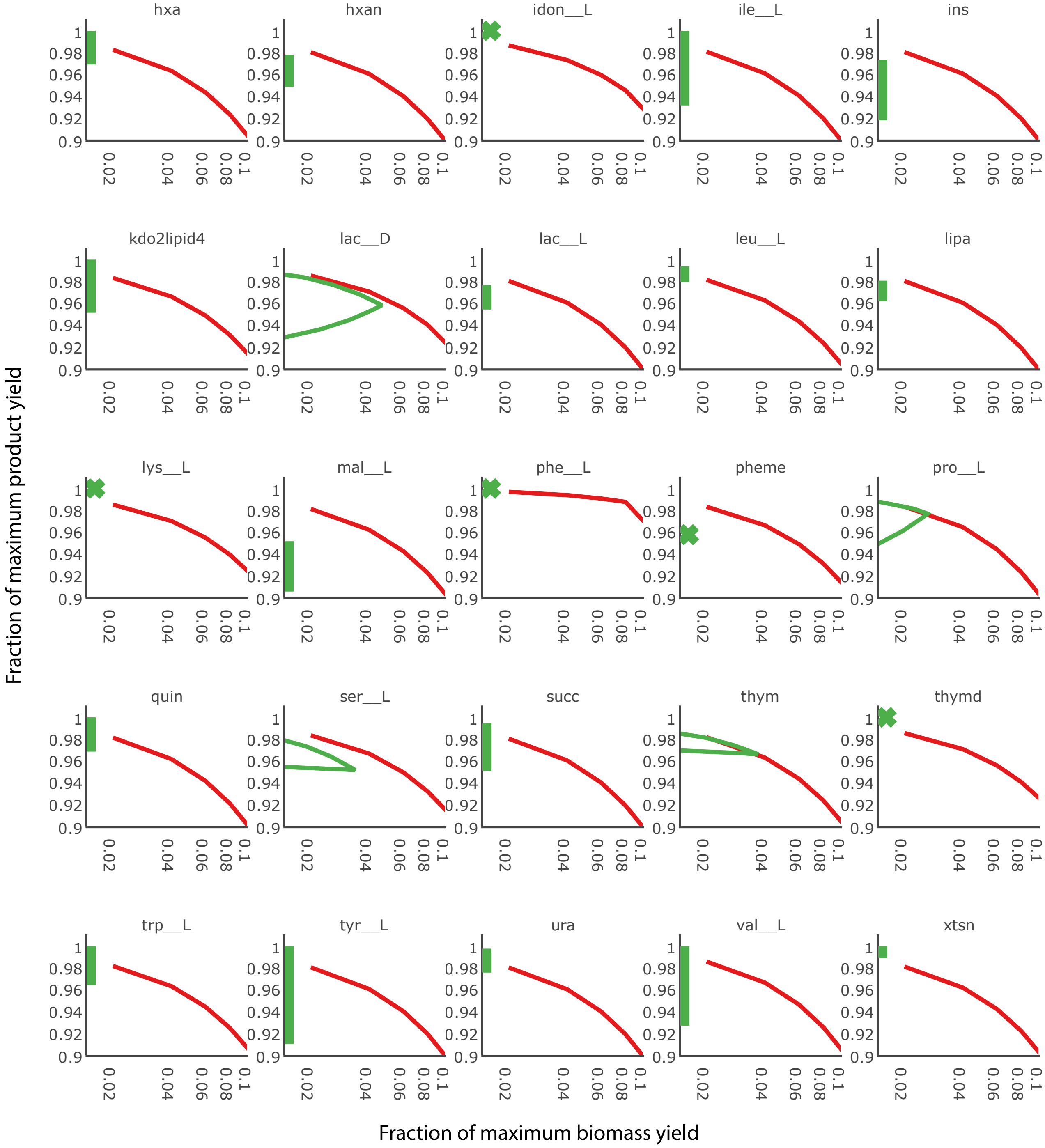

Supplementary Figure 3: Part 1. Production envelopes for fully decoupled strategies using single valves. Log-log axes are used to highlight features at low growth rates. Production states which have near-zero growth rate are displayed as a vertical line. Production states which have a negligible production flux variability are display as X. The growth state is shown in red and the production state is shown in green.

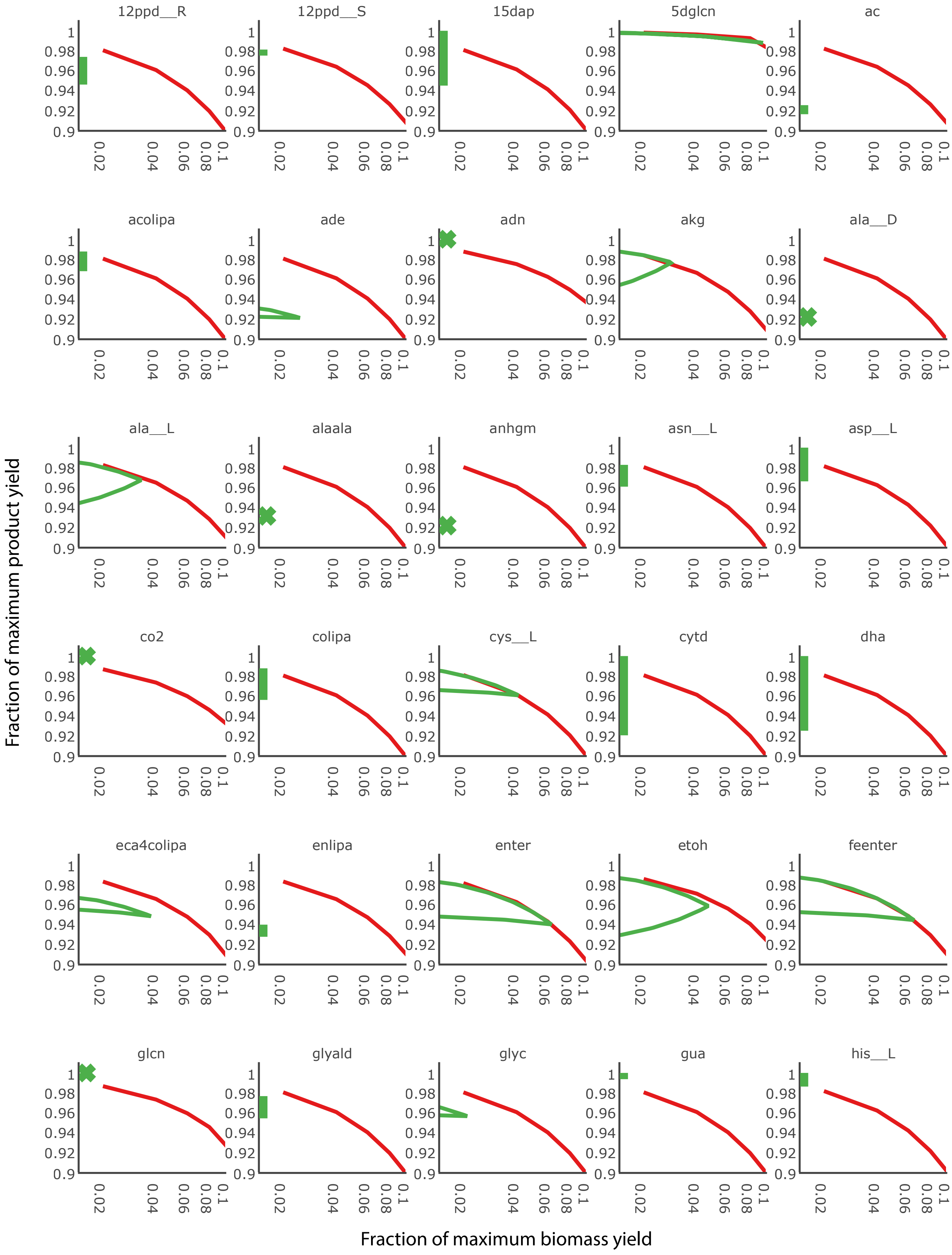

Supplementary Figure 4: Part 2. Production envelopes for fully decoupled strategies using single valves. Log-log axes are used to highlight features at low growth rates. Production states which have near-zero growth rate are displayed as a vertical line. Production states which have a negligible production flux variability are display as X. The growth state is shown in red and the production state is shown in green.

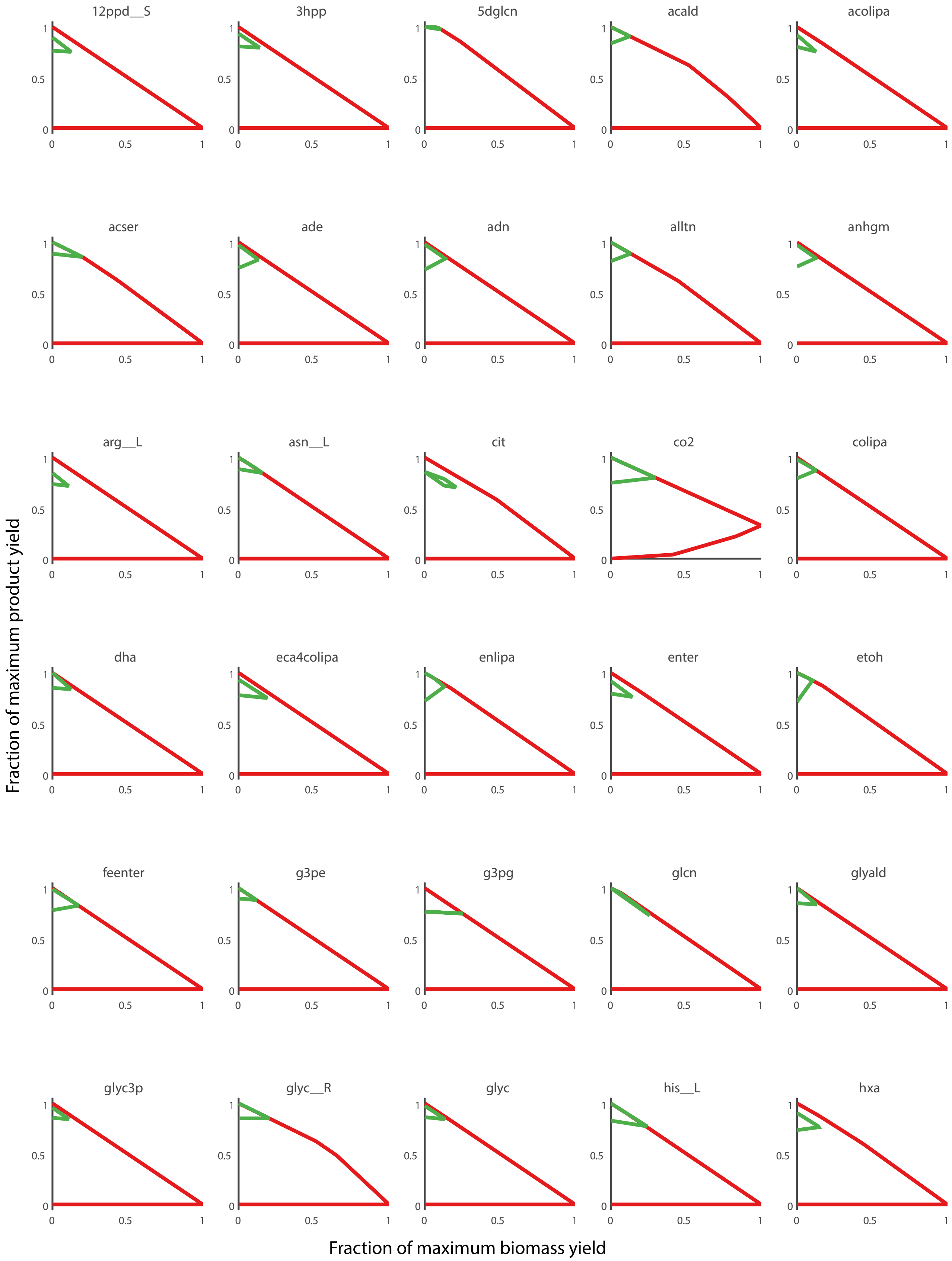

Supplementary Figure 5: Part 1. Production envelopes for partially decoupled strategies using single valves. The growth state is shown in red and the production state is shown in green.

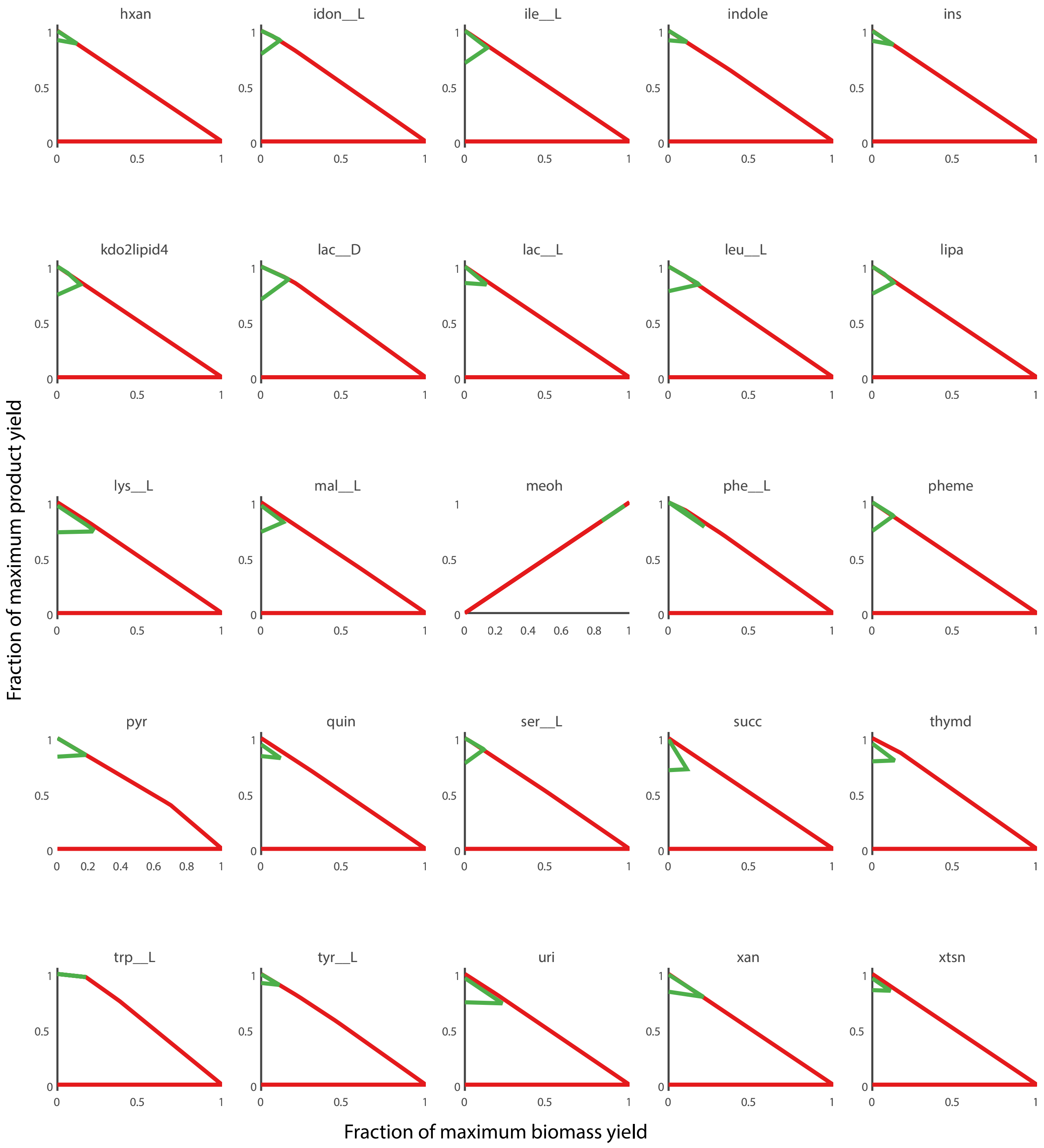

Supplementary Figure 6: Part 2. Production envelopes for partially decoupled strategies using single valves. The growth state is shown in red and the production state is shown in green. Note: methanol passed the initial screen; however, the low yield generally makes this product infeasible to produce from glucose.

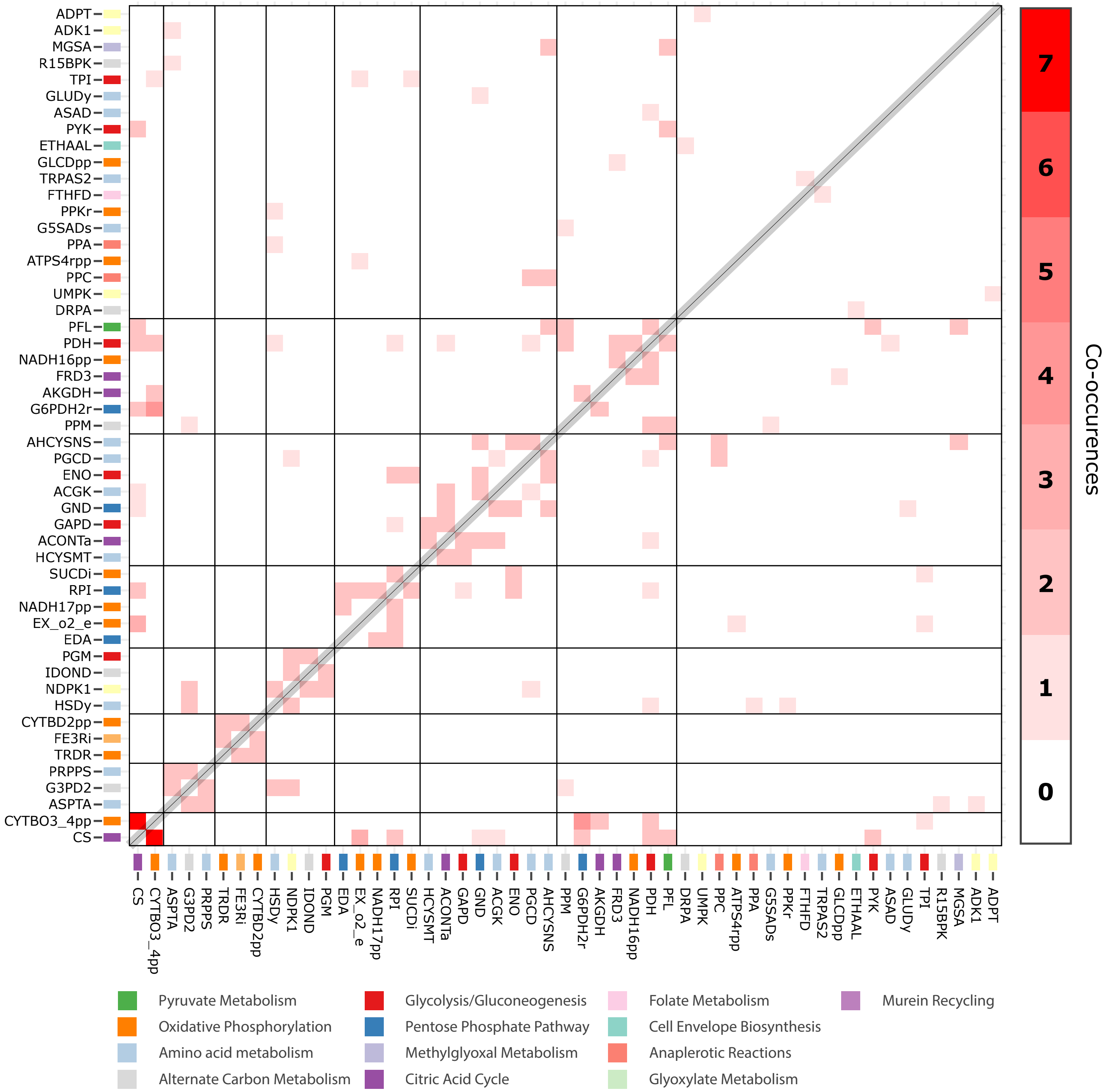

Supplementary Figure 7: Clustered heat maps of co-occurring valves for fully decoupled production. Double and triple valves were identified, and an adjacency was built from their co-occurences. This adjacency matrix was clustered using spectral clustering, and sorted by cluster density. Flux through these valves should be controlled in parallel to shift phenotypes between growth and production states.

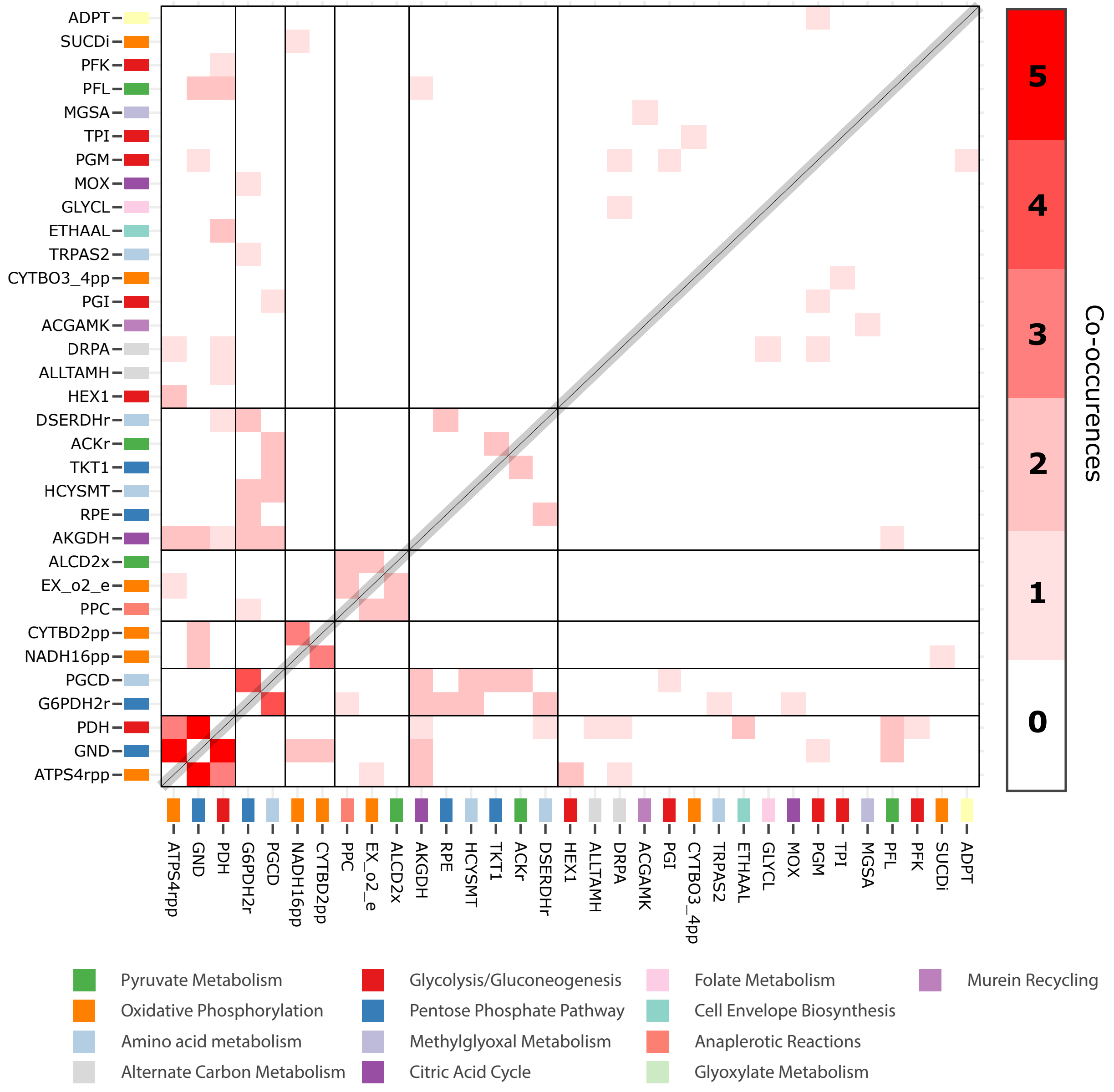

Supplementary Figure 8: Clustered heat maps of co-occurring valves for partially decoupled production. Double and triple valves were identified, and an adjacency was built from their co-occurences. This adjacency matrix was clustered using spectral clustering, and sorted by cluster density. Flux through these valves should be controlled in parallel to shift phenotypes between growth and production states.

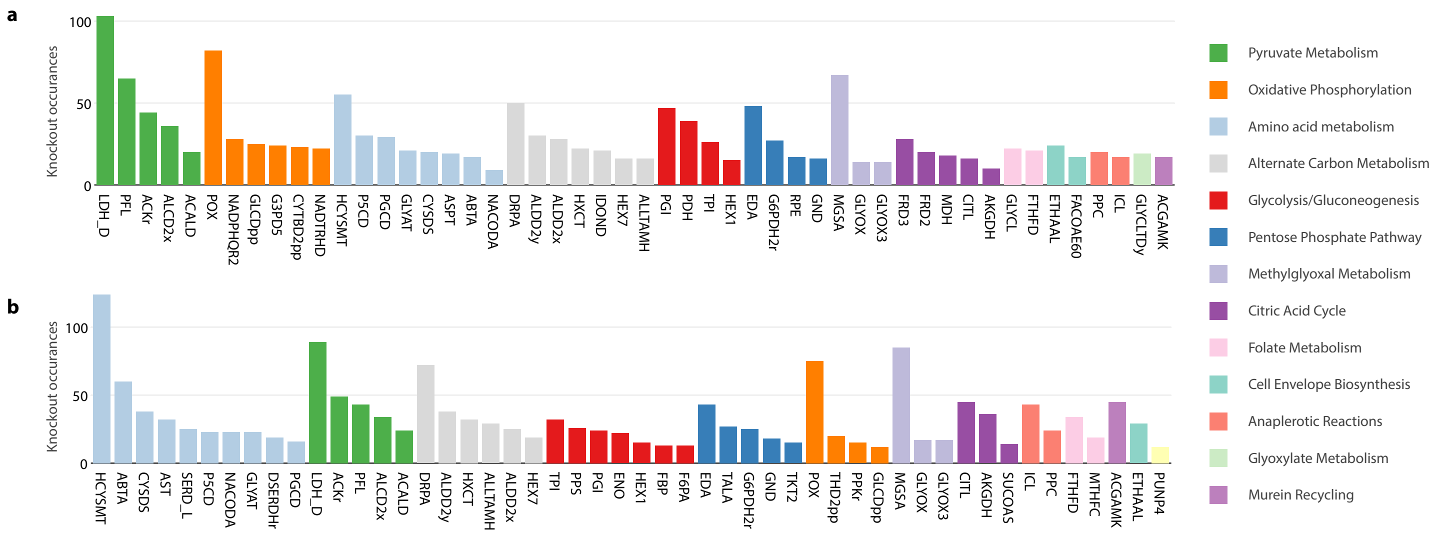

Supplementary Figure 9: Occurrences of each knockout in simulations targeting optimal valves, representing the sum of data from simulations for single, double and triple valve strategies. (a) Knockout occurrences for fully decoupled production. (b) Knockout occurrences for partially decoupled production strategies.

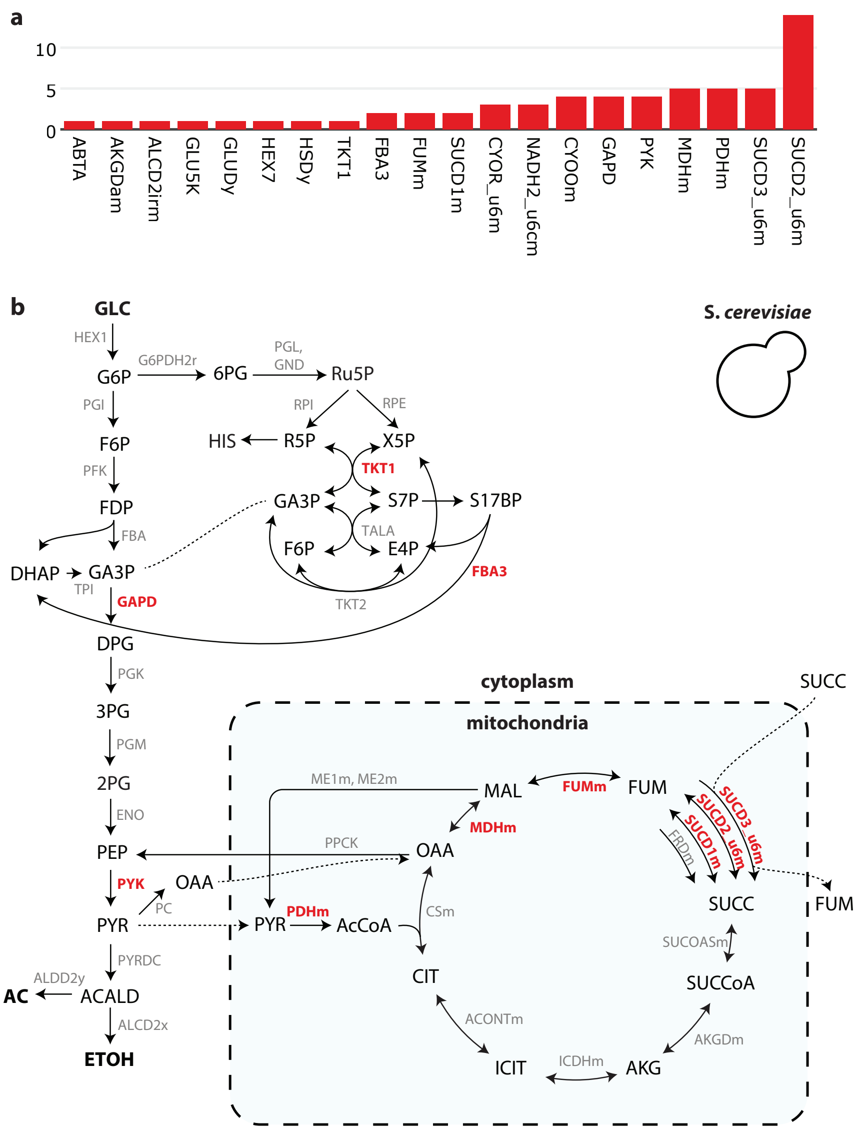

Supplementary Figure 10: Single valves identified in iMM904, a genome-scale metabolic model of *S. cerevisiae*. Strategies achieve over 70% of theoretical maximum product yield and a biomass yield of 0.001 gdw/mmol in the production state, and over 90% of theoretical maximum biomass yield in the growth state. (a) Valve occurences in strategies identified for 61 of 84 naturally producible metabolites from glucose. (b) Core metabolic map highlighting identified valves in red.

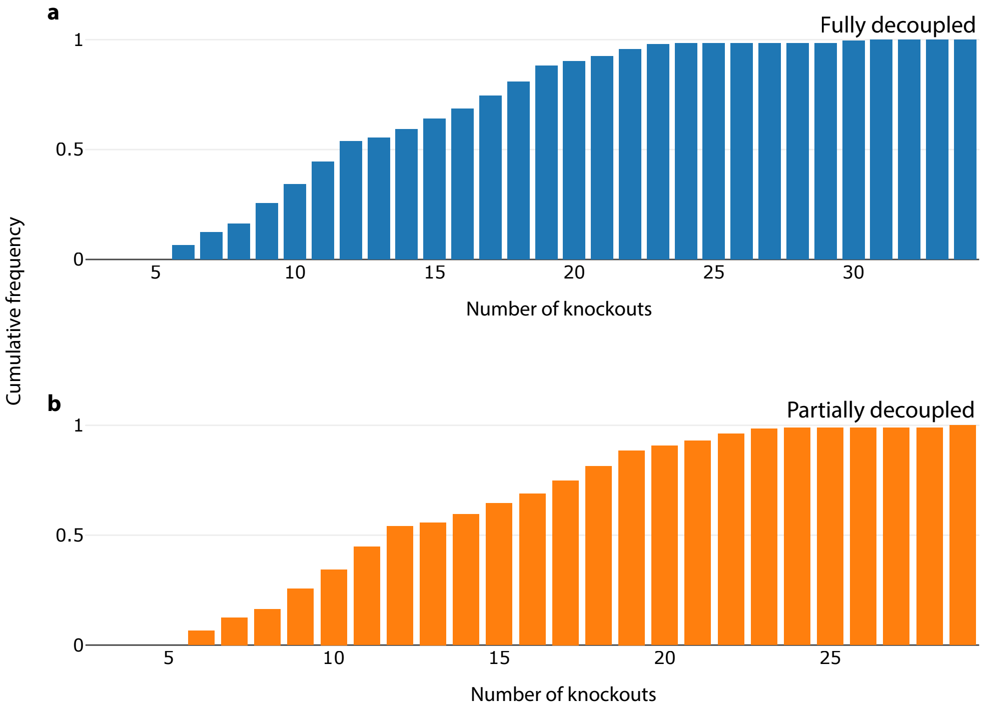

Supplementary Figure 11: Cumulative frequency distribution of required number of knockouts for (a) fully decoupled strategies and (b) partially decoupled strategies.

Supplementary Table 1: Targeted products in *E. coli* genome-scale model, associated reactions, and identified valves for both full and partial decoupling. Dash indicates no solution was found.

		**Identified valve**	
**Metabolite**	**Reaction**	**Full decoupling**	**Partial decoupling**
(R)-Propane-1,2-diol	EX_12ppd-R(e)	FRD3	—
(S)-Propane-1,2-diol	EX_12ppd-S(e)	CS	PGM
1,5-Diaminopentane	EX_15dap(e)	PFL	—
3-Hydroxypropanoate	EX_3hpp(e)	—	PGI
4-Aminobutanoate	EX_4abut(e)	—	—
5-Dehydro-D-gluconate	EX_5dglcn(e)	GAPD	PGM
L-alanine-D-glutamate-meso-2,6-diaminoheptanedioate	EX_LalaDgluMdap(e)	—	—
L-alanine-D-glutamate-meso-2,6-diaminoheptanedioate-D-alanine	EX_LalaDgluMdapDala(e)	—	—
Acetate	EX_ac(e)	EX_o2_e	—
Acetaldehyde	EX_acald(e)	—	EX_o2_e
4-Amino-4-deoxy-L-arabinose modified core oligosaccharide lipid A	EX_acolipa(e)	CS	PPC
O-Acetyl-L-serine	EX_acser(e)	—	DSERDHr
Adenine	EX_ade(e)	XYLI2	PGM
Adenosine	EX_adn(e)	PGM	G6PDH2r
Agmatine	EX_agm(e)	—	—
2-Oxoglutarate	EX_akg(e)	GND	—
D-Alanine	EX_ala-D(e)	R15BPK	—
L-Alanine	EX_ala-L(e)	EX_o2_e	—
D-Alanyl-D-alanine	EX_alaala(e)	NDPK1	—
Allantoin	EX_alltn(e)	—	PDH
N-Acetyl-D-glucosamine(anhydrous)N-Acetylmuramic acid	EX_anhgm(e)	GAPD	G6PDH2r
L-Arginine	EX_arg-L(e)	—	AKGDH
L-Asparagine	EX_asn-L(e)	CS	PGCD
L-Aspartate	EX_asp-L(e)	NDPK1	—
Cys-Gly	EX_cgly(e)	—	—
Citrate	EX_cit(e)	—	TPI
CO2	EX_co2(e)	GAPD	PGM
Core oligosaccharide lipid A	EX_colipa(e)	CS	TKT2
L-Cysteine	EX_cys-L(e)	PGM	—
Cytidine	EX_cytd(e)	PDH	—
Dihydroxyacetone	EX_dha(e)	GLUDy	PGM
(enterobacterial common antigen)x4 core oligosaccharide lipid A	EX_eca4colipa(e)	PPC	TALA
Phosphoethanolamine KDO(2)-lipid (A)	EX_enlipa(e)	ATPS4rpp	PGCD
Enterochelin	EX_enter(e)	TKT2	PGM

		Identified valve	
**Metabolite**	**Reaction**	**Full decoupling**	**Partial decoupling**
Ethanolamine	EX_etha(e)	—	—
Ethanol	EX_etoh(e)	EX_o2_e	EX_o2_e
Fe-enterobactin	EX_feenter(e)	EDA	TKT2
Formate	EX_for(e)	—	—
Sn-Glycero-3-phosphoethanolamine	EX_g3pe(e)	—	MGSA
Glycerophosphoglycerol	EX_g3pg(e)	—	TPI
D-Gluconate	EX_glcn(e)	GAPD	PDH
L-Glutamate	EX_glu-L(e)	—	—
Glycine	EX_gly(e)	—	—
D-Glyceraldehyde	EX_glyald(e)	GAPD	PGCD
Glycerol	EX_glyc(e)	GAPD	HEX7
(R)-Glycerate	EX_glyc-R(e)	—	GND
Glycerol 3-phosphate	EX_glyc3p(e)	—	PGM
Glycolate	EX_glyclt(e)	—	—
Reduced glutathione	EX_gthrd(e)	—	—
Guanine	EX_gua(e)	ENO	—
L-Histidine	EX_his-L(e)	PDH	TRPS2
L-Homoserine	EX_hom-L(e)	—	—
Hexanoate (n-C6:0)	EX_hxa(e)	EX_o2_e	EX_o2_e
Hypoxanthine	EX_hxan(e)	DRPA	PGM
L-Idonate	EX_idon-L(e)	GAPD	AKGDH
L-Isoleucine	EX_ile-L(e)	PDH	PDH
Indole	EX_indole(e)	—	F6PA
Inosine	EX_ins(e)	PDH	PGM
KDO(2)-lipid IV(A)	EX_kdo2lipid4(e)	CS	RPI
D-Lactate	EX_lac-D(e)	EX_o2_e	EX_o2_e
L-Lactate	EX_lac-L(e)	GAPD	PGCD
L-Leucine	EX_leu-L(e)	GND	AKGDH
KDO(2)-lipid (A)	EX_lipa(e)	CS	RPI
Cold adapted KDO(2)-lipid (A)	EX_lipa_cold(e)	—	—
L-Lysine	EX_lys-L(e)	CS	HEX1
L-Malate	EX_mal-L(e)	FRD2	PDH
Methanol	EX_meoh(e)	—	PYK
Ornithine	EX_orn(e)	—	—
L-Phenylalanine	EX_phe-L(e)	PPM	PDH
Protoheme	EX_pheme(e)	RPI	AKGDH
L-Proline	EX_pro-L(e)	MTHFC	—
Putrescine	EX_ptrc(e)	—	—
Pyruvate	EX_pyr(e)	—	PDH
Quinate	EX_quin(e)	PDH	DHAPT
L-Serine	EX_ser-L(e)	PGM	AKGDH
Succinate	EX_succ(e)	PPKr	EX_o2_e
L-Threonine	EX_thr-L(e)	—	—
Thymine	EX_thym(e)	TKT2	—
Thymidine	EX_thymd(e)	PDH	PGM
L-Tryptophan	EX_trp-L(e)	PDH	EDA
L-Tyrosine	EX_tyr-L(e)	CS	PGI
Uracil	EX_ura(e)	DRPA	—
Urea	EX_urea(e)	—	—

**Metabolite**	**Reaction**	**Identified valve**<br>**Full decoupling**	**Partial decoupling**
Uridine	EX_uri(e)	—	HEX7
L-Valine	EX_val-L(e)	EX_o2_e	—
Xanthine	EX_xan(e)	—	PGM
Xanthosine	EX_xtsn(e)	DRPA	TKT1

Supplementary Table 2: Targeted products in *S. cerevisiae* genome-scale model, associated reactions, and identified valves. Dash indicates no solution was found.

Metabolite	Reaction	Valve
2 Hydroxybutyrate C4H7O3	EX_2hb_e	NADDPp
2 methylbutyl acetate C7H14O2	EX_2mbac_e	–
2 methylbutyraldehyde C5H10O	EX_2mbald_e	–
2 methyl 1 butanol C5H12O	EX_2mbtoh_e	MDH
2 methylpropanal C4H8O	EX_2mppal_e	PDHm
2 phenylethanol C8H10O	EX_2phetoh_e	MDH
3-Carboxy-3-hydroxy-4-methylpentanoate	EX_3c3hmp_e	SUCD2_u6m
3 Methylbutanal C5H10O	EX_3mbald_e	SUCD3_u6m
(S)-3-Methyl-2-oxopentanoate	EX_3mop_e	–
4-Aminobutanoate	EX_4abut_e	SUCD2_u6m
4-Aminobenzoate	EX_4abz_e	PYK
N N bisformyl dityrosine C20H22N2O8	EX_Nbfortyr_e	–
Acetate	EX_ac_e	SUCD2_u6m
Acetaldehyde	EX_acald_e	SUCD2_u6m
Acetic ester C4H8O2	EX_aces_e	SUCD2_u6m
2-Oxoglutarate	EX_akg_e	FUMm
L-Alanine	EX_ala__L_e	CYOR_u6m
L-Arginine	EX_arg__L_e	–
L-Asparagine	EX_asn__L_e	–
L-Aspartate	EX_asp__L_e	–
R R 2 3 Butanediol C4H10O2	EX_btd_RR_e	CYOOm
Citrate	EX_cit_e	SUCD3_u6m
CO2 CO2	EX_co2_e	GAPD
L-Cysteine	EX_cys__L_e	MDH
DTTP C10H13N2O14P3	EX_dttp_e	–
Episterol C28H46O	EX_epist_e	–
Ergosterol C28H44O	EX_ergst_e	–
Ethanol	EX_etoh_e	CYOOm
Fecosterol C28H46O	EX_fecost_e	–
Formate	EX_for_e	FBA3
Fumarate	EX_fum_e	PDHm
Sn-Glycero-3-phosphocholine	EX_g3pc_e	GLPT
D-Glucosamine 6-phosphate	EX_gam6p_e	GAPD
L-Glutamine	EX_gln__L_e	FUMm
L-Glutamate	EX_glu__L_e	SUCD2_u6m
Glyoxylate	EX_glx_e	–
Glycine	EX_gly_e	–
Glycerol	EX_glyc_e	NADDPp
Guanine	EX_gua_e	–
H2O H2O	EX_h2o_e	PYK
H+	EX_h_e	–
L-Histidine	EX_his__L_e	HSDxi
Hypoxanthine	EX_hxan_e	–
Isoamyl acetate C7H14O2	EX_iamac_e	SUCD1m
Isoamyl alcohol C5H12O	EX_iamoh_e	SUCD3_u6m
Isobutyl acetate C6H12O2	EX_ibutac_e	SUCD2_u6m
Isobutyl alcohol C4H10O	EX_ibutoh_e	CYOOm
Indole 3 acetaldehyde C10H9NO	EX_id3acald_e	SUCD3_u6m
L-Isoleucine	EX_ile__L_e	–
Indole 3 ethanol C10H11NO	EX_ind3eth_e	ALCD2x_copy2

Metabolite	Reaction	Valve
D-Lactate	EX_lac__D_e	GAPD
L-Lactate	EX_lac__L_e	GAPD
Lanosterol C30H50O	EX_lanost_e	SUCD1m
L-Leucine	EX_leu__L_e	SUCD3_u6m
L-Lysine	EX_lys__L_e	SUCD2_u6m
L-Malate	EX_mal__L_e	PDHm
L-Methionine	EX_met__L_e	GLUDxi
Nicotinamide adenine dinucleotide phosphate	EX_nadp_e	AKGDbm
Ammonium	EX_nh4_e	–
Oxaloacetate	EX_oaa_e	PDHm
Ornithine	EX_orn_e	NADDPp
Phenylacetaldehyde	EX_pacald_e	PYK
Adenosine 3',5'-bisphosphate	EX_pap_e	TKT1
L-Phenylalanine	EX_phe__L_e	–
Phenethyl acetate C10H12O2	EX_pheac_e	SUCD2_u6m
Phosphate	EX_pi_e	–
(R)-Pantothenate	EX_pnto__R_e	ABTt
L-Proline	EX_pro__L_e	SUCD2_u6m
Putrescine	EX_ptrc_e	SUCD2_u6m
Pyruvate	EX_pyr_e	SUCD2_u6m
D-Sorbitol	EX_sbt__D_e	CYOR_u6m
L-Serine	EX_ser__L_e	PDHm
Sulfite	EX_so3_e	FBA3
Spermidine	EX_spmd_e	MDH
Succinate	EX_succ_e	CYOR_u6m
L-Threonine	EX_thr__L_e	HEX4
Thymine C5H6N2O2	EX_thym_e	–
L-Tryptophan	EX_trp__L_e	PYK
Tetradecanoate (n-C14:0)	EX_ttdca_e	MDH
L-Tyrosine	EX_tyr__L_e	SUCD2_u6m
Urea CH4N2O	EX_urea_e	–
L-Valine	EX_val__L_e	CYOOm
Xanthine	EX_xan_e	–
Zymosterol C27H44O	EX_zymst_e	SUCD2_u6m
