# Supplementary material for: MoVE identifies metabolic valves to switch between phenotypic states
Source: Nat Commun. 2018 Dec 14;9:5332. doi: 10.1038/s41467-018-07719-4 (PMC6294006; doi:10.1038/s41467-018-07719-4)
Supplement: Supplementary file 3 — Description of Additional Supplementary Files [file 41467_2018_7719_MOESM3_ESM.pdf]

Description of Additional Supplementary Files

File Name: Supplementary Software 1
Description: Matlab implementation of the MoVE algorithm and example run scripts

File Name: Supplementary Data 1
Description: Summary of simulation results (valves)

File Name: Supplementary Data 2
Description: Raw results (knockouts and valves) of all simulations performed

File Name: Supplementary Data 3
Description: Python-readable (pickle serialized) data

File Name: Supplementary Data 4
Description: Reactions which were knocked out (upper and lower bound set to 0) for simulations to improve computational efficiency
